# The Perspectives of Individuals with Chronic Stroke on Motor Recovery: A Qualitative Analysis

**DOI:** 10.3390/healthcare12151523

**Published:** 2024-07-31

**Authors:** Amelia Cain, Carolee J. Winstein, Marika Demers

**Affiliations:** 1 Division of Biokinesiology and Physical Therapy, Herman Ostrow School of Dentistry, University of Southern California, Los Angeles, CA 90033, USA; winstein@usc.edu; 2 Department of Neurology, Keck School of Medicine, University of Southern California, Los Angeles, CA 90033, USA; 3 École de Réadaptation, Université de Montréal, Montreal, QC H3N 1X7, Canada; marika.demers@umontreal.ca; 4 Institut Universitaire de Réadaptation en Déficience Physique de Montréal, Centre for Interdisciplinary Research in Rehabilitation of Greater Montreal, CIUSSS Centre-Sud-de-l’Ile de Montréal, Montreal, QC H3S 1M9, Canada

**Keywords:** stroke, qualitative, mindset, motor recovery, patient perceptions, neurorehabilitation

## Abstract

The priorities of individuals with chronic stroke are not always reflected in clinical practice. This study provides insight into meaningful factors related to long-term motor recovery in stroke survivors. Thirty individuals with chronic stroke participated in semi-structured interviews about movement, recovery, and barriers to and facilitators of mobility and paretic arm use. The interviews were analyzed using inductive thematic analysis. Three categories, the individual, environment, and task, defined five emergent themes. Individual: (1) mindset is a strong and consistent influencer of daily physical activity and overall recovery; (2) severe physical impairment limits physical activity and recovery, regardless of other factors; and (3) a negative perception of disability impacts mindset and willingness to move in public. Environment: (4) social and physical environments influence physical activity and recovery. Task: (5) participation in meaningful activities increases physical activity and promotes long-term recovery. Strategies to incorporate paretic arm use, exercise, and encouragement from others facilitate physical activity. Insufficient paretic limb function, environmental obstacles, and fear are barriers to physical activity. Neurorehabilitation must address the factors that are meaningful to stroke survivors. Building motor capacity is essential and must be integrated with factors such as a positive mindset and proper environment. Individual differences reinforce the need for personalized care.

## 1. Introduction

Stroke remains the leading cause of long-term disability in the United States [1]. About 80% of stroke survivors experience motor impairment, which is directly related to reduced functional ability [2,3,4] and quality of life [5]. Recovery after stroke is complex and influenced by demographics, lesion size and location, extent of impairment, therapeutic interventions, genetic factors, social context, confidence, and more [6,7,8,9]. Motor recovery requires both improving the motor capacity and translating that capacity into daily activities that improve quality of life. 

Although clinicians and researchers recognize these complex contributors to motor recovery, the factors that are meaningful to individuals with chronic stroke for long-term motor recovery remain poorly understood. Clinician and patient priorities do not always align, which may contribute to the neglect of outcomes that are most meaningful to the patient [10,11,12]. A qualitative study comparing the points of view of stroke patients, caregivers, and physical therapists in an inpatient rehabilitation center found that stroke survivors’ most common priority for their post-stroke recovery focused on activity and participation, while physical therapists prioritized body functions and structures [10]. Current rehabilitation interventions may prioritize improving capacity over activity- and participation-based goals, which may be more important to the patient. Unfortunately, evidence suggests that improving motor capacity does not inherently translate to improvements in daily activity [13]. To directly improve outcomes that are meaningful to patients, we must first understand patient perspectives and develop interventions that address their priorities.

Recent work emphasizes the need to better understand patient perspectives [14,15,16] and to manage stroke as a long-term, chronic condition [17]. Qualitative methods provide otherwise inaccessible insight to understand patient perspectives on factors that influence long-term recovery and to develop interventions that align with patients’ priorities. Prior qualitative work has focused on specific aspects of stroke recovery, such as adherence to rehabilitation [18], community reintegration [19], participation [20], and paretic arm use [21]. The qualitative analysis of detailed interviews in this study provides unique, comprehensive insight into the perspectives of stroke survivors on their overall movement behavior and long-term recovery. The perspectives of individuals in the chronic phase after stroke may differ from their initial priorities in the acute and sub-acute phases when healthcare support is more prevalent. Within this context, recovery refers to a person’s functional recovery, which is conceptualized as a dynamic integration of biopsychosocial factors, rather than their neurologic recovery [22]. 

This study aims to (1) understand stroke survivors’ perceptions about the key factors that impact their daily movement behavior and long-term recovery, and (2) provide recommendations to integrate these factors into patient-centered neurorehabilitation.

## 2. Materials and Methods

### 2.1. Study Design

We used a qualitative grounded theory design [23] to construct theory related to post-stroke motor behavior and recovery from the analyzed data. We collected qualitative data as part of a larger parent study, the RESTORE (wearables for stroke function in the natural environment) observational study [24]. The consolidated criteria for reporting qualitative research (COREQ) checklist was followed to optimize the rigor of reporting [25].

### 2.2. Participants

Thirty-two chronic stroke survivors were purposefully recruited on a face-to-face basis or via telephone or email through the IRB-approved Registry for Aging and Rehabilitation Evaluation (RARE) Database. Participants were included if they had sustained ischemic or hemorrhagic stroke, were >18 years of age, lived at home, and could communicate in English. The exclusion criteria included unilateral spatial neglect (positive score on 2/3 screening measures), required assistance for ambulation, severe cognitive or language impairments prohibiting communication with the interviewer, or other medical conditions with potential to interfere with participation. All the participants were informed of the procedures and requirements for participation and provided informed consent. The study complies with the Declaration of Helsinki. The study procedures were approved by the IRB at the University of Southern California (HS 19-00984 and HS 20-00015).

### 2.3. Procedures

The semi-structured interviews were led by M.D., who has extensive experience interviewing, and assisted by A.C., who was trained in interview techniques. The interviews were conducted in person or via video call, per participant preference. Communication strategies were used to accommodate participants with aphasia or hearing impairments. Participants’ family members or other research staff were occasionally present during the interview. The interviews were structured around a detailed interview guide (see the Supplementary Material) on the barriers to and facilitators of physical activity (PA), factors influencing paretic arm use and mobility, and motor recovery. At the end of each interview, the interviewer summarized her understanding of the participants’ meaning behind the key points. Participants were asked to reflect on the summary and add or clarify any missing or misrepresented information as an initial method of achieving member reflections [26]. The transcripts were not returned to the participants for member checking since this is no longer considered an effective method for validation [26,27]. The interviews were audio-recorded and transcribed verbatim. Field notes were written after each interview. No repeat interviews were required.

### 2.4. Data Analysis

Two clinician-researchers, A.C. and M.D, independently analyzed the transcripts using reflexive, inductive thematic analysis with a codebook approach. The process of the thematic analysis was modeled after Braun and Clarke’s framework [28,29], and it was performed using the NVivo 12 software (QSR International Pty Ltd., Melbourne, Australia). After data familiarization, a preliminary codebook was developed, with definitions for each code. Coding was initiated after the first five participants were analyzed, and it continued until data saturation was reached, as determined by no new codes emerging. Themes were developed, reviewed, refined, defined, and named [28,29]. 

The codes and resulting themes were data- rather than theory-driven, meaning they were developed based on the generated ideas from participants’ interviews and theory was then used to interpret and assign meaning to the data [27]. The researchers reached collaborative consensus on codes and themes throughout the analysis. Any disagreements were discussed until consensus was reached, with a third reviewer (C.J.W.) contributing to the discussion to interpret data, resolve disagreements, and refine themes. An audit trail was kept for the rationale behind the decision-making. Once the thematic analysis was completed, content analysis based on the code frequency or number of participants who referenced specific codes or themes was applied for the purpose of internal validity [30] and demonstration that our analysis reflected the data. Interview data extracts are included to illustrate the findings in the Results section [31].

### 2.5. Research Team and Reflexivity

The analysis team is composed of women with a variety of theoretical backgrounds, which inherently influence the qualitative analysis and results. M.D., OT, PhD, is an assistant professor with experience conducting qualitative research on the perspectives of stroke survivors, with projects related to young stroke survivors and the feasibility of rehabilitation interventions. C.J.W., PT, PhD, is a professor with clinical experience as a physical therapist who has contributed extensively to neurological rehabilitation research, with an emphasis on stroke survivors’ motor behavior. A.C., PT, DPT, is a neurologic clinical specialist in physical therapy. Several participants had professional relationships with the interviewer through prior research studies. Participants had limited knowledge of the researchers’ personal backgrounds and goals but were provided with information regarding the purpose and procedures of the RESTORE study, consistent with obtaining informed consent. We did not anticipate that subjects’ prior participation in research studies would negatively impact the data collection. However, it may have influenced willingness to participate or may have encouraged participants to share insightful perspectives they may not have otherwise shared with a stranger. Participants’ perceptions of the researchers’ positionality, such as their age, gender, or social class, may also have impacted the data collection [32].

To optimize reflexivity, the analysis team reflected throughout on the contributions of individuals’ backgrounds and potential biases to the analysis [33]. To further promote rigor, an individual independent of the analysis team recruited the participants, the researchers refrained from judgement during the interviews or analysis, and thematic analysis was performed iteratively. The relevant literature was not reviewed until after the thematic analysis was nearly completed, with only the step of manuscript writing remaining, to reduce the influence that outside theory might have on the analysis.

## 3. Results

Thirty chronic stroke survivors participated (see Table 1 for the participants’ characteristics). Participants averaged 7.6 years post-stroke, a mean age of 58.6 (SD 13.1) years, with moderate-to-severe arm motor impairment (average Fugl-Meyer Assessment Upper Extremity score 41.2/66), and most were independent with ambulation (60% with Functional Ambulation Category of 5). Eight participants had aphasia, identified by an NIHSS Best Language score greater than zero. Two recruited participants were not included after we determined they met the exclusion criteria; this resulted in thirty enrolled participants. None dropped out. Each interview lasted, on average, 21.0 (SD 9.4) min.

**Figure 1:**
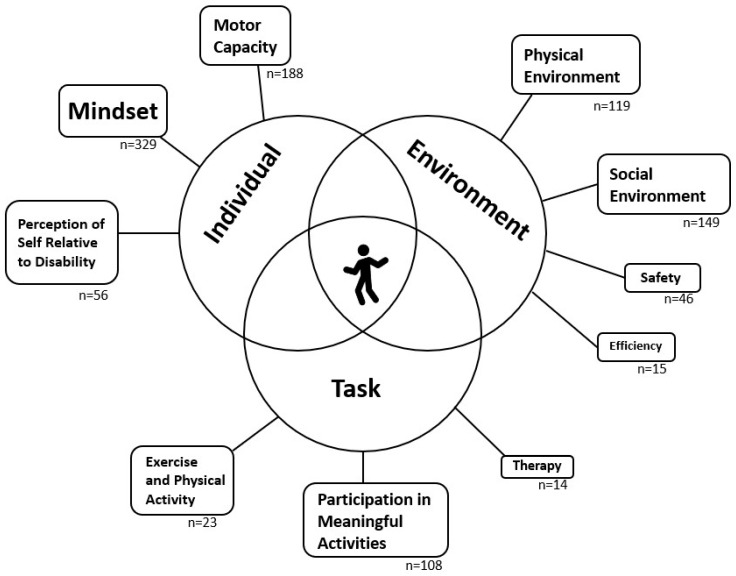
Thematic map of factors influencing movement behavior and recovery after stroke. Thematic map representing the main themes and sub-themes identified by chronic stroke survivors as related to factors influencing movement behavior and recovery. These themes can be described in the context of the overlapping domains of the individual, environment, and task, consistent with modern motor control theory. The relative font size of each theme represents the importance of the theme as determined by thematic and content analysis. The code frequency is noted below each theme or sub-theme.

The five themes that emerged were classified based on the interaction of three overlapping categories: the individual, the environment, and tasks (see Figure 1). The major themes related to the individual are as follows: (1) mindset is a strong and consistent influencer of daily physical activity (PA) and overall recovery; (2) severe physical impairment limits PA and recovery, regardless of other factors; and (3) a negative perception of disability impacts mindset and willingness to move in public. Specific to the environment, (4) social and physical environments influence PA and recovery. Finally, the major theme related to tasks is as follows: (5) participation in meaningful activities increases PA and promotes long-term recovery. Stroke survivors identified mindset, motor capacity, environment, perception of disability, and participation in meaningful activities as important factors influencing movement behavior and post-stroke recovery. Within these themes, participants identified facilitators of and barriers to PA (see Table 2 for examples).

### 3.1. The Individual

#### 3.1.1. Theme 1: Mindset Is a Strong and Consistent Influencer of Daily Physical Activity and Overall Recovery

This theme encompasses the role of mindset, i.e., the individual’s attitude or beliefs that determine their response to and interpretation of situations, in terms of movement behavior and long-term recovery. All the participants discussed mindset, with a particular emphasis on the importance of internal motivation. Independence, self-efficacy, fear, persistence, goal-setting, psychological health, and faith were additional notable aspects of mindset that influence recovery. Mindset was most often identified as a positive factor supporting recovery and encompassing individual characteristics like determination, attitude, or stubbornness. People reported having set goals and challenges for themselves and worked hard to achieve them.

P12: What do I think influenced the most of my recovery of my walking? (laughs) Oh, determination? (…) I had all the volition in the world to get better and I wasn’t satisfied. (…) Hard work, will, determination.

P22: I’m very stubborn. If there’s a way to do it, I’ll figure it out.

P18: We never, never give up and never really accept that you can’t do anything. You just keep pushing forward. (…) I’m very motivated in that sense.

A few participants believed that their mindset has held them back from achieving full recovery, identifying the barriers of fear and low confidence.

P43: I’m seeing a counselor to help overcome the fear of falling. And I think that’s probably my biggest inhibitor.

#### 3.1.2. Theme 2: Severe Physical Impairment Limits Physical Activity and Recovery, Regardless of Other Factors

Unsurprisingly, the participants reported that ongoing physical impairments (e.g., pain, edema, weakness, etc.) reduced their ability to engage in desired movements. Reduced motor capacity limited their participation in meaningful activities, regardless of motivation or effort.

P33: Because my hand is not functional enough to do a lot of things, my arm doesn’t get used.

P18: My left arm unfortunately is not really—I really can’t use it for a lot because of its condition.

P15: I try. I try but I can’t.

#### 3.1.3. Theme 3: Negative Perception of Disability Impacts Mindset and Willingness to Move in Public Places

The majority (n = 19) of participants felt discouraged or frustrated by their perceptions of being disabled or having others view them as disabled. Some described their desire to “be normal” as a motivator for increased PA; however, many expressed feeling frustrated or insecure due to their disability. Consequently, individuals with stroke changed the way they moved in public. Participants expressed challenges with body image, describing their movements as robotic or drunk, and being discouraged by gained weight or changes in muscle tone. A few harnessed their challenges with body image as motivators to increase their PA; however, most described being self-conscious and reducing their activity in social environments.

P12: I don’t like the way I look when I walk. I don’t like the speed that I walk with. I just did whatever I had to do, strengthening, whatever to improve it.

P33: I walk funny when I see myself in the mirror and I walk—it’s clear, when people stare at me, and they wonder what’s wrong. (…) I feel like a slug, and that bothers me a lot.

P10: I don’t see the need for people to see me handicapped.

The participants endorsed adapting their movement in response to varying environmental contexts. While most agreed on certain aspects of the environment that impact PA, such as being less active when it is dark or raining, individuals had diverse perspectives on which factors are enablers versus barriers. Some participants viewed walking outdoors as enjoyable or a pleasant challenge, while others viewed walking outdoors as unsafe. Safety concerns were a frequently cited barrier to PA.

P39: When I’m inside I feel safer, especially at home. Whereas when you are outside, you run into the society. Maybe there are rushing kids or something. That’s when you really have to be careful what you are doing.

P10: When I take a first step, when I go outside, I naturally get a smile on my face like. Like wow I’m going to do this.

Similarly, some individuals cited the support of friends and family as vital in increasing their PA and enhancing their recovery, while others reported that a lack of social support forced them to be more independent and hastened their recovery. A few expressed a desire for more social support to gain confidence in taking on challenges. The need for efficiency in certain social environments (e.g., keeping pace with a partner or attending a meeting on time) caused individuals to reduce their paretic arm use and attention to gait quality. Overall, the participants agreed that the social environment was a strong influencer of PA and recovery, but the perspectives were varied regarding what was considered a barrier to versus a facilitator of PA.

P31: My mother is one thing. She [walks] every day. So, I have to walk with her.

P22: My grandson said you have to play handball. Well, I’m going to figure out how to not fall and play handball.

P46: If I had someone who could walk around with me, I would try [to walk] a little bit farther.

P05: I also think living by myself, because—because seeing people who have a spouse or people who were living by themselves, I find that people living by themselves improved faster, because they were forced to.

### 3.2. The Task—Theme 5: Participation in Meaningful Activities Increases Physical Activity and Promotes Long-Term Recovery

The participants reported that they want to engage in the same activities they enjoyed prior to their stroke. Most (n = 17) described the importance of independence with daily activities, which caused them to increase their paretic arm use and mobility to successfully complete those activities. The participants found strategies to remember to incorporate their paretic arm in daily activities; otherwise, they would forget to try. Obligations outside the home such as work, volunteering, appointments, or social groups provided opportunities for increased PA. Recreational activities, exercise, therapy, and caring for pets were viewed as meaningful activities that facilitated movement and recovery.

P31: I like to play [video]games, and I’m going to play these games no matter how much it takes.

P18: I’m very lucky that I was always very active before my stroke, and then when I had the stroke, because of the volunteer activities that I did, kept me very active and I learned a lot.

P17: [I] sweep the whole floor in the hallway and then I mopped. So, it was a lot of work yesterday, but you know, I don’t look at it as work, I look at it as therapy or exercise and (…) so I’m good with that.

P37: --having my dog, because I have to walk her. I don’t have to, of course, I could get somebody else to do it, but it gives me motivation to walk.

### 3.3. Content Analysis

**Figure 2:**
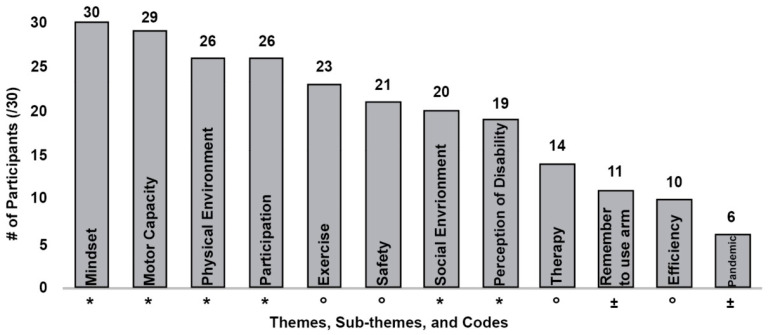
Number of participants referencing common themes (*), sub-themes (°), and codes (±).

The content analysis results support our thematic analysis. Figure 2 plots the number of participants who discussed the main themes, sub-themes, and codes, demonstrating that more than two-thirds identified mindset, motor capacity, environment, and participation in meaningful activities as factors influencing their PA and recovery. Additionally, the most frequently referenced themes were mindset (reference frequency, n = 329), motor capacity (n = 188), social environment (n = 149), physical environment (n = 119), participation in meaningful activities (n = 108), and perception of disability (n = 56). The frequency of the references to each code did not notably differ between those with mild to moderate impairment and those with more severe motor impairment.

## 4. Discussion

Five overlapping themes emerged and were related to modern motor control theory, which recognizes the influence of the individual, environment, and task on PA. Our participants most strongly emphasized the influence of mindset on PA and recovery, frequently citing aspects of mindset such as internal motivation, self-efficacy, and fear. Motivation enhances motor learning [34,35], and higher motivation is associated with increased PA and participation [36,37]. A recent analysis of stroke survivors’ tweets also recognized motivation as one of the most referenced themes related to stroke recovery [38]. Our results also well supplement quantitative evidence indicating that self-efficacy can be a powerful predictor of functional independence and a moderator of the effects of rehabilitation [39], particularly impacting the translation of rehabilitation into real-world performance [9,40]. Mindset, which encompasses both self-efficacy and motivation, impacts treatment efficacy [41,42,43,44,45,46,47]. Importantly, an individual’s mindset can be influenced, particularly by the relationship between patient and clinician, suggesting an opportunity to harness this influence to improve neurorehabilitation efficacy [48,49,50].

Our results align with previous work focused on more specific aspects of stroke recovery, such as adherence to rehabilitation [18], community reintegration [19], participation [20], or paretic arm use [21]. These studies also identified the roles of mindset, social support, familiar physical environments, and participation in meaningful, tailored therapeutic activities as facilitators of adherence [18], reintegration [19], or participation [20]. Limited motor capacity, related to our second most referenced theme, is generally recognized as a barrier to exercise adherence [18] or arm use [21]. Our results expand upon the referenced prior work by providing a more comprehensive lens on the meaningfulness of these factors to movement and motor recovery after stroke. Motor capacity, mindset, social support, physical environment, and participation in meaningful therapeutic activities are important to individuals with chronic stroke, not only in the specific contexts of exercise adherence or limb use but also more broadly in their long-term movement behaviors and overall recovery.

Our participants, particularly those with severe physical impairment, stated that without adequate capacity, other factors impacting recovery were irrelevant. This indicates that improving upon motor capacity is essential. However, improved motor capacity alone is insufficient to achieve recovery when considering the restoration of an individuals’ activity and participation [13,51]. Rehabilitation must address motor capacity in combination with other influential factors, such as mindset and environment, to achieve meaningful outcomes [52]. 

Although most participants emphasized the influence of both the social and physical environments on PA and recovery, there were noteworthy individual differences in perceptions of facilitators of versus barriers to PA. This perceptual conundrum is consistent with the qualitative literature exploring paretic arm use [21]. Our results align with those of Twardzik et al. (2022) [53], who report that outdoor environments require vigilance and result in reduced ability and willingness to be active outside of the home [53]. Most participants acknowledged the influence that their perception of disability had on their mindset and willingness to move in public. However, like the influence of the environment, there were individual differences in whether a negative perception of disability enhanced or limited long-term motor recovery. These individual differences highlight the need for personalized, collaborative approaches to care to overcome environmental barriers.

The identified themes reinforce the integrated motor control theory presented by Shumway-Cook and Woollacott in *Motor Control: Integrating Research into Clinical Practice* [54]. This theory integrates elements of prior (ecological, hierarchical, systems, and dynamic systems) theories to describe movement to be generated by an individual to meet the demands of a specific task in a specific environment [54]. Movement, therefore, is not a result of either the individual, task, or environment, but rather the interaction between these domains. The perspectives of individuals with chronic stroke as portrayed in this study further emphasize the need to simultaneously address the individual, environment, and task, as opposed to prioritizing a single domain, to promote long-term motor recovery.

Despite the increased focus on providing valuable, patient-centered care, the majority of stroke survivors continue to report long-term unmet needs [15,55]. To achieve durable and meaningful recovery, researchers and clinicians must understand what stroke survivors consider to be important influencers of their movement behavior and long-term recovery. The generated themes related to mindset, capacity, environment, perceptions of disability and participation in meaningful activities inherently interact in their contribution to long-term recovery. The emergent themes and the facilitators of and barriers to PA can promote patient-centered interventions designed to specifically address factors most meaningful to stroke survivors.

### 4.1. Strategies to Integrate Patient Perspectives into Neurorehabilitation: Current Possibilities and Future Directions

Our results support the more contemporary idea that rehabilitation programs may be most effective when they target both motor capacity and mindset [40,56,57]. There are several potential strategies for addressing mindset in rehabilitation research and care, such as interventions to enhance motivation or self-efficacy [58]. Although each individual is motivated differently, there are four theoretical contributors to self-efficacy: performance accomplishments, vicarious experience, verbal persuasion, and emotional arousal [59]. Successful performance of challenging activities, interaction with other stroke survivors, encouragement from friends or health providers, and stress reduction are examples of strategies to build task-specific self-efficacy. More research is needed to understand which aspects of mindset are most impactful for recovery, whether these aspects are consistent across stroke survivors, and whether interventions intended to target mindset impact rehabilitation outcomes. 

A promising line of research using behavioral economics, a field that combines economics and psychology to explain decision-making, models strategies for integrating factors such as motivation and social context into rehabilitation to improve health behaviors [60]. Because health behavior change is highly complex, insights from behavioral economics may inform care and future research initiatives. Additionally, rehabilitation is best achieved through interdisciplinary treatment to adequately address the complexities of motor behavior after stroke. For example, psychological care may be critical to address the identified barriers related to fear or the influence of the negative perceptions of others. Rehabilitation psychologists should be integrated into the clinical and research teams to better consider stroke survivors’ moods and motivations and prioritize a holistic conceptualization of treatment [61]. 

**Figure 3:**
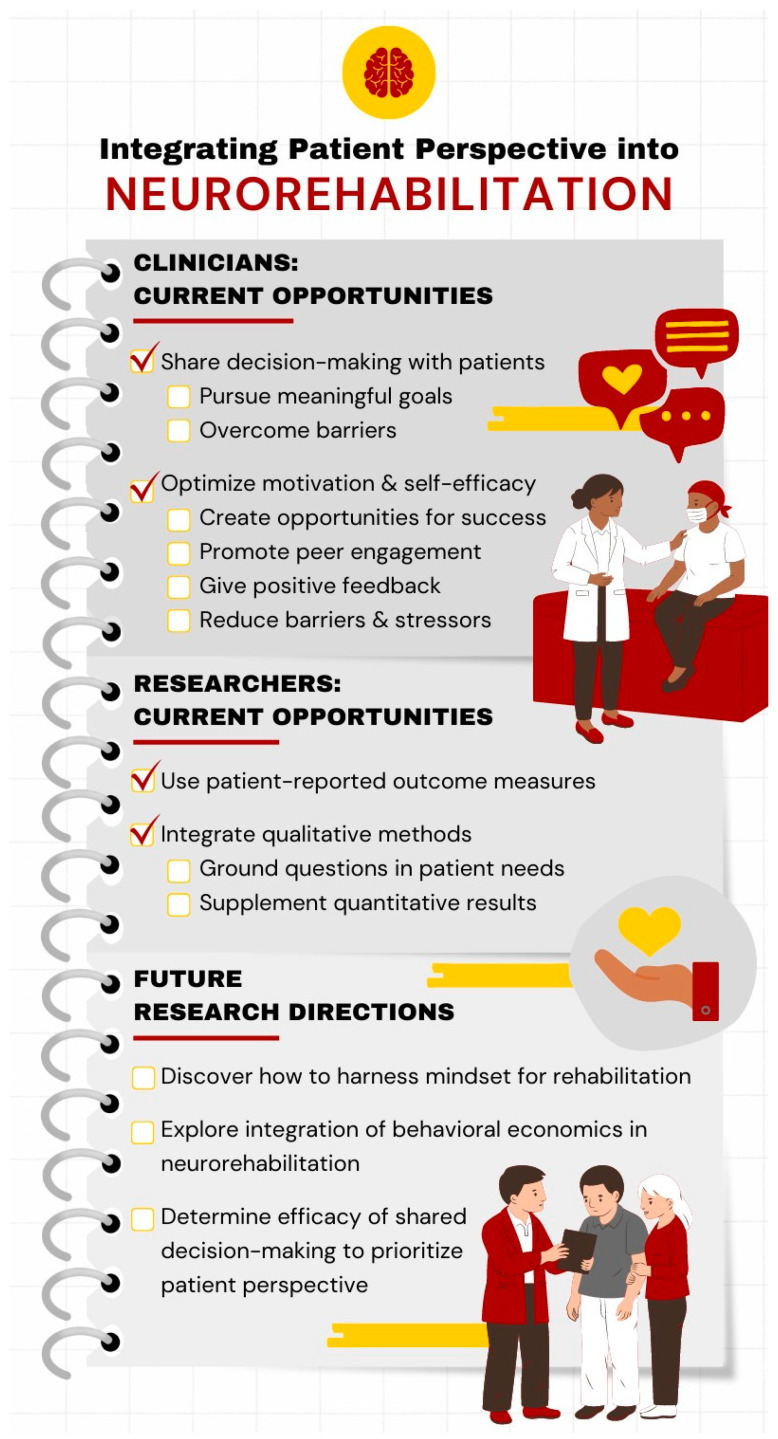
Strategies to integrate the perspectives of individuals with stroke into neurorehabilitation practice and research.

In clinical practice, shared decision-making between the patient and the clinician is one strategy to improve patients’ understanding and satisfaction, and to align approaches with patients’ values [58,62,63]. Shared decision-making may also help to identify and address individual barriers to recovery, such as reduced social support or environmental obstacles. Additional current clinical strategies to promote the integration of patient perspectives are to engage in motivational interviewing and the development of the therapeutic alliance, although additional research is needed to determine the efficacy of these strategies [64,65]. In research, valuing patient-reported outcomes and qualitative data as a necessary supplement to objective quantitative measures will help to better understand participants’ perspectives on recovery-supportive interventions and ground research questions in needs that are relevant to our patients [15] (see Figure 3 for a summary of the strategies to integrate patient perspectives into neurorehabilitation).

### 4.2. Limitations

We purposefully recruited a diverse group of chronic stroke survivors, including participants with aphasia and a wide range of stroke chronicity and motor impairment; however, all the participants were independent ambulators. Our findings can thus not be generalized to those with severe mobility deficits. The percentages of participants identifying as each race are comparable to the population percentages in Los Angeles, CA, where the study was conducted [66]. However, the percentage of Hispanic participants is not representative of the population or observed stroke rates [67]. The reduced percentage of Hispanic participants may limit the representativeness of these findings to Hispanic individuals post-stroke. Additionally, we specifically asked participants about the factors contributing to their recovery; however, we did not have participants define recovery for themselves and participants likely have varying perspectives on what recovery means to them. 

Given that this study was specifically conducted in chronic stroke survivors, the identified themes may differ from the priorities that would be expressed in the acute or sub-acute period. The included recommendations to integrate patient perspectives through shared decision-making and optimizing self-efficacy and motivation remain relevant in the acute or sub-acute period; however, clinicians should prioritize the perspectives of the individual being treated in their current state. Because healthcare support often becomes less prevalent in the chronic phases, the perspectives provided in this study should be considered in reference to and anticipation of stroke survivors’ long-term goals.

Although our use of content analysis was primarily for internal validity, quantifying qualitative data can be problematic. It can detract from the meaning derived through qualitative analysis and instead focus on quantitative aspects such as word/code frequency, which can misrepresent the significance of participants’ statements [68]. For example, the frequency of codes referencing mindset is the highest compared to the other themes and sub-themes, but this may be attributed to mindset being broader, with more opportunity to code statements as related to this topic. Therefore, the results of the content analysis should be regarded only as supplemental to the thematic findings.

## 5. Conclusions

To achieve meaningful, durable recovery post-stroke, neurorehabilitation research and practice must address the factors chronic stroke survivors consider to be important. Comprehensive patient-centered care must consider each individual’s unique mindset, motor capacity, physical and social environments, perception of disability, and participation in meaningful activities.

## Figures and Tables

**Table 1:** Participants’ characteristics.

Characteristic	Mean ± SD or %
Gender (%)	Men: 60.0 Women: 36.7 Non-binary: 3.3
Age (years)	58.6 ± 13.1
Race (%)	American Indian or Alaska Native: 0 Asian: 16.7 Black: 16.7 Native Hawaiian or Pacific Islander: 10 White or Caucasian: 36.7 More than one Race: 16.7 Not reported/unknown: 3.3
Ethnicity (%)	Hispanic: 30.0 Non-Hispanic: 70.0
Time since stroke (years)	7.6 ± 4.5 (range: 1.0–21.2)
Hemisphere affected by the stroke (%)	Left: 60.0 Right: 40.0
Dominance of arm affected by stroke (%)	Dominant: 53.3 Non-Dominant: 46.7
National Institutes of Health Stroke Scale—Best Language (%)	No aphasia: 73.3 Aphasia: 26.6
Montreal Cognitive Assessment (/30)	24.7 ± 3.4
Fugl-Meyer Assessment Upper Extremity (/66)	41.2 ± 18.0 (Range: 18–66)
Functional ambulation category (%)	3 (supervision): 13.3 4 (independent, level surfaces): 26.7 5 (independent, all surfaces): 60.0
10-m walk test (m/s)	Self-paced: 0.75 ± 0.40 Fast-paced: 0.96 ± 0.53
Use of assistive device (%)	Yes: 66.67 No: 33.33

**Table 2:** Perceived enablers of and barriers to physical activity and recovery.

Positive Factors			Limiting Factors		
Enabler	Representative Quote	N	Barrier	Representative Quote	N
Strategies to incorporate paretic arm	P18: “really, it’s whatever task I’m working on. I mean, I (…) I basically live alone so I do everything myself. You know, when I’m folding clothes or when I’m cooking, I use tools to help me where I’m typically just using my [unaffected] arm, but when I’m doing other kinds of tasks, if I’m gluing something or if I’m repairing a device, I will use my [affected] arm to steady it.	20	Physical obstacles in the environment	P46: “I don’t like going up and down on sidewalks or anything like that. I don’t feel comfortable”.	24
Positive mindset	P29: “So as far as what motivates me, I tell myself I have to do better every day. And even if you don’t see end results every day, I tell myself a week later was better than it was a week before”.	25	Lack of function	P02: “[sigh] If my arm is good, I’ll do everything, but I can’t use it now”.	15
Support and encouragements from family and peers	P09: “[My daughter] would tell me, “Mom, you’re overextending your knee. Mom, try to walk better.” So when I will walk to the restroom, I’ll be like, “Use your leg”, “left, right, left, right, left, right,” and then, and then I’ll just start doing it over by myself.”	21	Fear	P39: “It’s more of the fear of maybe getting bumped into or falling or something. That’s when we need to be even more careful”.	11
Therapeutic approaches and exercise	P17: “I think exercise, the gym. That’s what gets me tired, but when I recuperate from the tiredness, I see the difference, I can see improvements. I would say the exercise is what helps me the most”.	19	Perception of others	P10: “Like when I’m around people […] I get conscious, you know, I try to act like I’m normal”.	8
Desire for independence	P35: “I don’t want anybody to have to push me in a wheelchair and wash my face and nothing. I want to be independent as long as I can”.	16	Lack of social support	P35: “I think things have changed because of my disability. I don’t think people realize that there’s such a thing as social exclusion”.	7

## Data Availability

Dataset available on request from the authors.

## Supplementary Materials

The following supporting information can be downloaded at https://www.mdpi.com/article/10.3390/healthcare12151523/s1, File S1: Interview guide excerpt.
