# Supplementary material for: The Perspectives of Individuals with Chronic Stroke on Motor Recovery: A Qualitative Analysis
Source: Healthcare (Basel). 2024 Jul 31;12(15):1523. doi: 10.3390/healthcare12151523 (PMC11312011; doi:10.3390/healthcare12151523)
Supplement: Supplementary file 1 [file healthcare-12-01523-s001.zip › healthcare-3127112-File S1-supplementary.pdf]

Wea**R**abl**E**s for **S**troke Func**TiO**n in the Natu**R**al **E**nvironment

Interview guide (Excerpt)

Introduction:
Thank you again for your participation in this study. We want to know your opinion about what influences you to use your most-affected arm in your daily activities and what influences you to move around your house or in the community.

You should try to say your honest and true opinion. Your feedback will help us to design an intervention that can be used in the rehabilitation for stroke survivors like you. The discussion will take no more than 60 minutes/ 1 hour. May I record the discussion to facilitate its recollection? (if yes, switch on the recorder)

Confidentiality:
Despite being taped, I would like to assure you that the discussion will be confidential. The recording will be transferred to a password-protected computer until it is transcribed word for word. Then, the recording will be destroyed.

2. Arm use in daily activities

How much are you using your most-affected arm in your everyday activities?
What are their main problems you have with using your arm?
Please describe the activities you think are difficult for you to accomplish because of your arm?
What motivates you to use your more-affected arm to do your everyday activities?
What do you think influenced your motor recovery?
What helps you to use your most-affected arm more?
What limits you to use your arm since your stroke?

> Please describe if there was anything that influenced how much you used your arm <u>after</u> your rehabilitation.

3. Walking behavior in daily activities

Thinking about the amount of walking activity you do ***inside your home***, how does it compare to before you had your stroke?

- Do you feel you are more active, less active or about the same?

Now thinking about the amount of walking activity you do ***outside of your home***, how does it compare to before you had your stroke?

- Do you feel you are more active, less active or about the same?

What motivates you to walk in your everyday activities?
What would you say are the main problems or limitations that you have when you walk?
What do you do to overcome these problems or limitations when you walk?
What would motivate you to walk more?
What barriers prevent you from walking more?
